# Predictors of psychological well-being during imposed prolonged absence from work

**DOI:** 10.1186/s12982-025-01056-w

**Published:** 2025-11-04

**Authors:** Holly Blake, Juliet Hassard, Maria Karanika-Murray, Wei Hoong Choo, Louise Thomson

**Affiliations:** 1 School of Health Sciences, University of Nottingham, Nottingham, UK; 2 NIHR Nottingham Biomedical Research Centre, Nottingham, UK; 3 Queen’s Business School, Queen’s University Belfast, Belfast, UK; 4 School of Management, University of Leicester, Leicester, UK; 5 School of Medicine, University of Nottingham, Nottingham, UK; 6 Institute of Mental Health, Nottingham, UK

**Keywords:** Workforce, Wellbeing, Furlough, Survey, Work-related absence, Job uncertainty

## Abstract

**Background:**

Between March 2020 and September 2021, 11.7 million employee jobs were furloughed through the UK Coronavirus Job Retention Scheme (JRS). Imposed work absence shielded workers from job loss, but furloughed workers had increased risk of poor mental health compared to those who stayed working. Understanding the factors that mitigate psychological distress during imposed work absence can inform actions to be taken in future crises.

**Aims:**

To explore the relationships between (a) work and home demands with well-being outcomes, and (b) personal and organisational resources with well-being outcomes, during periods of imposed prolonged absence and uncertainty.

**Methods:**

We analysed online survey data collected with furloughed workers in the UK ‘Wellbeing of the Workforce Study’. Measures included psychological well-being, anxiety, life satisfaction, job insecurity, home demands (quantitative and emotional), organisational support for work-family balance, and personal resources (resilience, purpose, and coping ability).

**Results:**

Psychological well-being was associated positively with quantitative home demands (β = 0.24, *p* < 0.05) and personal resources (β = 0.45, *p* < 0.001). Life satisfaction was associated negatively with emotional demands at home (β = –0.26, *p* < 0.05) and positively with personal resources (β = 0.30, *p* < 0.05). Perceived job insecurity was positively associated with anxiety (β = 0.36, *p* < 0.001).

**Conclusions:**

Job-related factors are less influential during periods of employment uncertainty compared to personal and home resources. Decision-makers should provide psychological support during periods of job uncertainty and bolster the essential benefits of personal and home resources. Moving forwards, these findings may have broader applicability to other challenges and crises, such as suspension from work, or role changes resulting from organisational restructuring.

## Introduction

Conditions of imposed prolonged absence and uncertainty, as experienced by furloughed workers during the COVID-19 pandemic, can help us to better understand psychological well-being outcomes and factors associated with such outcomes. Between March 2020 and September 2021, 11.7 million employee jobs were furloughed through the UK Coronavirus Job Retention Scheme (JRS), providing grants to employers so they could retain and continue to pay staff up to 80% of their wages during pandemic-related lockdowns [1]. The JRS shielded employees from job loss but introduced new psychological and social challenges through imposed prolonged absence and ensuing uncertainty.

Although the JRS mitigated psychological distress in furloughed workers compared to those who became unemployed [2, 3], workers furloughed had poorer mental health and lower life satisfaction compared to those who remained working [2]. The furlough scheme provided a natural experiment by which to examine the unique effects of imposed absence from work. Understanding the factors that mitigate psychological distress during imposed work absence will inform recommendations for employee support during times of extreme uncertainty (e.g., furlough, suspension, organisational restructuring).

Crisis situations challenge our understanding of human behaviour in naturalistic situations. A crisis presents a risk and uncertainty that requires an immediate response [4]: it evokes different negative emotions, linked with different appraisals, which in turn trigger different action tendencies. The deciding factor between engagement and withdrawal is locus control - for example, anger, regret, or guilt will be linked to an appraisal of control and a tendency engage, whereas fear, disappointment, or shame will be linked to lack of control and withdrawal [4].

Crisis situations may also reduce some types of demands or restrict access to specific types of resources. Applied to workplace crises, the Job Demands-Resources model proposes that demands and resources at different levels (e.g., individual, family, organisational) will interact with each other [5]. In the case of imposed absence (furlough, detention, etc.), the loss of access to and control of job-related demands and resources will render non-work factors more salient. Thus, crisis situations may make non-work demands more salient and heighten a risk response tendency to protect resources that are perceived to be within one’s control [6], i.e., personal as opposed to job-related resources. We aimed to explore the relationship between (a) work and home demands and well-being outcomes, and (b) personal and organisational resources and well-being outcomes during periods of imposed absence in a sample of furloughed workers.

## Materials and methods

We analysed data from 98 furloughed participants in the UK ‘Well-being of the Workforce’ (WoW) longitudinal cohort study, completing an online survey between April and June 2020. Participants were 79% female (39%:<40 years, 60%:>40 years), 92% White, and 31% had caregiving responsibilities. Full procedures for the WoW study are published elsewhere [7]. Ethical approval was obtained from the University of Nottingham Research Ethics Committee (Ref: 03-0420). The survey was promoted via social media and professional networks. Potential participants accessed an online information sheet which explained the purpose of the study, procedures, data storage and how long the survey took to complete, at the end of which they clicked to provide their online consent. Participants were informed that by voluntarily completing and submitting the online survey they were providing their written informed consent to take part, after which they could access the survey questions.

Psychological well-being was measured using the WHO-5 Well-being Index [8], a 5-item measure with items rated on a 6-point scale (0=“At no time”, 5=“All of the time”). Higher scores indicated better well-being. Overall satisfaction with life was measured using a single item [9] on a 5-point scale (0=“Very satisfied”, 4=“Very dissatisfied”). Anxiety was measured using the 7-item Generalised Anxiety Disorder Scale (GAD-7 [10]), with responses rated on a 4-point scale (0=“Not at all”; 3=“Nearly every day”). Higher scores indicated greater anxiety.

Job insecurity was assessed using a single item from the Copenhagen Psychosocial Questionnaire [11], capturing concerns about job stability (i.e., worry about future employability), rated on a 5-point scale (0 = “Never”, 4 = “Always”). Demands at home was measured using the Home Demands Scale (adapted from [12]), including subscales capturing quantitative (task-based) and emotional (affective) demands. Subscales comprised three items rated on a 5-point scale (0=“Always”, 4=“Never”). The degree to which employees felt supported by their organisation in balancing work and family responsibilities was measured using the 10-item Perceived Organizational Family Support Scale (POFS [13]), with items rated on a 5-point scale (0=“Never”, 4=“Always”). Higher scores indicated greater perceived support. Personal resources were measured with the 4-item Conservation of Resources Evaluation (COR-E [14]) scale, covering resilience, purpose, and coping ability, with items rated on a 5-point scale (0=“Not at all”, 4=“To a great degree”). Higher scores indicate greater personal resources.

Data were analysed using IBM SPSS Statistics for Windows version 26. Analysis included Pearson’s product-moment correlation and hierarchical stepwise regression.

## Results

We present descriptive statistics and bivariate correlations (Table 1) and results of regression analyses (Table 2).

**Table 1:** Correlation matrix and descriptive statistics for study variables

		Mean (SD)	Range	1	2	3	4	5	6	7	8	9
1	Psychological well-being	48.58 (20.41)	0–100	α = 0.87								
2	Life satisfaction	2.42 (0.91)	0–4	0.63**	α=/							
3	Anxiety symptoms	7.85 (4.96)	0–21	− 0.64**	− 0.56**	α = 0.90						
4	Job insecurity	7.69 (3.44)	0–12	− 0.32**	− 0.34**	0.46**	α=/					
5	Facilitation of acceptance	18.77 (8.84)	0–36	0.16	0.29**	− 0.25*	− 0.28**	α = 0.90				
6	Perceived organisational family support	16.72 (11.55)	0–40	0.03	0.21*	− 0.14	− 0.07	0.59**	α = 0.97			
7	Quantitative home demands	6.72 (2.50)	0–12	0.18	0.16	− 0.09	0.12	0.05	0.01	α = 0.75		
8	Emotional home demands	5.72 (2.41)	0–12	− 0.17	− 0.19	0.21*	0.29**	0.01	0.05	0.40**	α = 0.66	
9	Personal resources	8.27 (4.03)	0–16	0.51**	0.42**	− 0.24*	− 0.22*	0.14	0.13	− 0.01	− 0.12	α = 0.77

Correlation is significant at the 0.01 level (2 tailed), * Correlation is significant at the 0.05 level (2 tailed), brackets = Cronbach’s alpha, *N* = 98

**Table 2:** Summary of hierarchical regression analyses for variables predicting psychological well-being, anxiety symptoms and life satisfaction (*N* = 98)

Step	Predictor		Psychological well-being (*n* = 98)						Anxiety symptoms (*n* = 98)						Satisfaction with life (*n* = 98)				
			B	SE(B)	Bias	Upper CI	Lower CI		B	SE(B)	Bias	Upper CI	Lower CI		B	SE(B)	Bias	Upper CI	Lower CI
Step 1																			
Covariates	Age		− 0.28	0.15	–	0.03	− 0.563		0.03	0.04	0.00	0.11	− 0.05		0.00	0.00	0.00	0.02	− 0.01
	Gender		6.08	4.71	0.38	15.98	− 2.86		− 0.87	1.04	− 0.04	1.15	−2.99		0.08	0.20	–	0.509	− 0.34
	Ethnicity		− 0.85	3.63	− 0.77	4.61	− 11.64		0.76	1.25	0.09	3.03	−1.40		− 0.10	0.18	0.02	0.28	− 0.40
	Caring Responsibility		2.99	3.90	0.01	10.20	− 4.32		0.24	1.21	0.073	2.95	−2.10		0.29	0.23	0.01	0.74	− 0.15
	∆R^2^	0.04						0.02						0.04					
Step 2																			
Work stressors	Job Insecurity		− 0.78	0.67	− 0.10	0.40	− 2.25		0.51**	0.15	0.00	0.78	0.24		− 0.05	0.03	− 0.0	0.00	− 0.11
	Facilitation of Acceptance		0.32	0.28	− 0.02	0.86	− 0.25		− 0.07	0.07	–	0.07	− 0.21		0.01	0.01	0.00	0.04	− 0.01
	∆R^2^	0.12*						0.23*						0.15*					
Step 3																			
Home stressors	Quantitative Home Demands		1.90*	0.79	0.05	3.44	0.38		− 0.39	0.20	0.00	0.01	− 0.81		0.09	0.05	0.00	0.17	− 00
	Emotional Home Demands		−1.708	1.089	− 0.073	0.30	−3.96		0.36	0.23	0.02	0.84	− 0.06		− 0.10*	0.04	− 0.01	− 0.02	− 0.19
	∆R^2^	0.07*						0.04						0.07*					
Step 4																			
Work and personal resources	Perceived Organisational Family Support		− 0.10	0.19	0.02	0.30	− 0.45		− 0.02	0.05	− 0.01	0.07	− 0.14		0.01	0.01	0.00	0.023	− 0.008
	Personal Resource		2.24**	0.39	− 0.03	2.93	1.42		− 0.16	0.10	0.00	0.03	− 0.37		0.07*	0.02	− 0.00	0.11	0.02
	∆R^2^	0.18**						0.02						0.09*					
	(Constant)		42.43	17.61	4.01				3.36	5.85	− 0.60				2.07	0.84	− 0.04		

The model predicting psychological well-being was statistically significant (*F* (10, 82) = 5.66, *p* < 0.001) accounting for 33.6% of the variance in scores. Quantitative home demands were positively associated with well-being (β = 0.24, *p* < 0.05). Personal resources were strongly associated with well-being (β = 0.45, *p* < 0.001), with higher personal resources linked to better psychological outcomes.

The model for life satisfaction was significant (*F* (10, 82) = 4.50, *p* < 0.001), explaining 27.6% of the variance in scores. Higher emotional demands at home (e.g., stress from caregiving or household conflicts) were negatively associated with life satisfaction (β = 0.26, *p* < 0.05). Personal resources were linked to higher life satisfaction (β = 0.30, *p* < 0.05).

The model for anxiety was significant (*F* (10, 80) = 3.61, *p* < 0.01), explaining 22.1% of the variance in scores. Job insecurity was positively associated with anxiety (β = 0.36, *p* < 0.001).

## Discussion

**Fig. 1:**
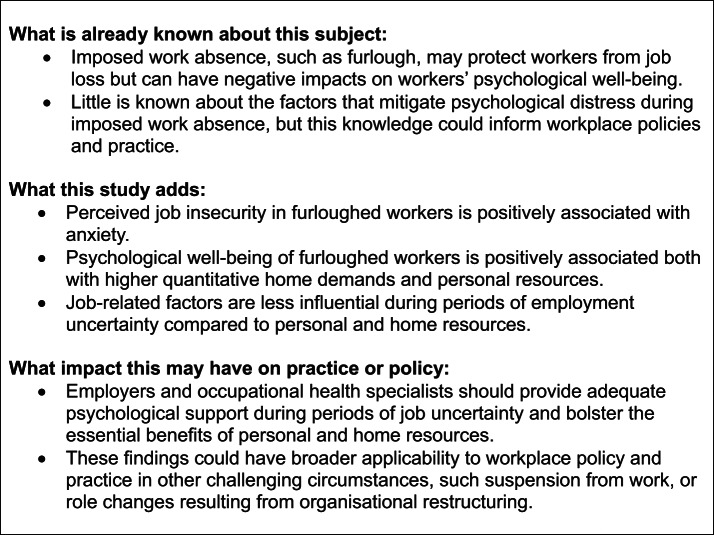
Context, findings and learning points

In this study, we explored the relationship between (a) work and home demands with well-being outcomes, and (b) personal and organisational resources with well-being outcomes, during furlough, a period of imposed prolonged absence and uncertainty. We analysed online survey data collected in the UK ‘Wellbeing of the Workforce Study’ with furloughed workers. Key findings are that personal and home resources were more influential to furloughed workers’ well-being outcomes than job-related factors. This is in line with decision-making under crisis theory [4] but not aligned with an expanded job demands-resources model in crisis situations [5]. Findings could apply to other challenging circumstances involving imposed work absence. The study context, key findings and learning points are shown in Fig. 1.

Overall, psychological well-being is strongly linked to personal resources and quantitative home demands, suggesting that maintaining routine and accessing internal coping resources are critical for mental health during times of imposed absence and uncertainty. Home responsibilities may provide structure and purpose, possibly offsetting the loss of routine caused by absence from work. Supporting the Conservation of Resources Theory [15], individuals with stronger personal resources (specifically resilience, purpose, and coping ability) may deal better with imposed absence.

Life satisfaction is linked to reduced emotional home demands and increased personal resources, indicating that while emotional stress at home during furlough may erode life satisfaction, strong personal skills for coping and adaptation can enhance it.

Anxiety in furloughed employees is primarily linked to increased job insecurity, highlighting the importance of employment stability for mental health during uncertainty. This aligns with research showing that anticipatory stress about economic security is a predominant factor driving disparities in psychological distress [16].

To our knowledge, this research is the first to show that essential contributors to psychological well-being under such circumstances are quantitative (but not emotional) home demands and personal resources (resilience, purpose, coping ability). Such factors appear to be more relevant to well-being outcomes than job-related factors during periods of furlough and employment uncertainty.

Study limitations include small sample size with respondents from one region in the UK, lack of causality and potential for self-report bias [17]. Respondents were predominantly White and female, although national data [18] show that women were more likely to be furloughed than men, and White workers were more likely to be furloughed than other ethnic groups, such as Asian workers.

Future research should aim to integrate job design and decision-making theory - crisis situations offer a fruitful way to challenge knowledge developed in naturalistic settings. Future studies could investigate the dynamics we observed across different industries and job roles, examine the effects of imposed prolonged absence from work under other circumstances than furlough (e.g., suspension from duties), and develop methods for effective subsequent return-to-work.

Based on these study findings, we advocate that decision-makers make provisions for psychological support in polices relating to absence from work mandatory. Support for employees during imposed prolonged work absence and uncertainty (e.g., furlough, suspension, organisational restructuring) should focus primarily on personal and home resources, and less on job-related factors. The power of personal and home resources may inform interventions which mitigate the adverse effects of imposed work absence on psychological well-being.

## Data Availability

The dataset for the UK Wellbeing of the Workforce study is available from the Nottingham Research Data Management Repository (http://doi.org/10.17639/nott.7435).
